# The Second No. of the Register

**Published:** 1847-10

**Authors:** 


### *The Second No. of the Register.*

It will be seen that Dr. E. Taylor has commenced the first No. of a series of articles on the operation of filling Teeth. They will run through the first volume. Dr. Ide, in the second No., will commence a series of articles on full setts of Teeth and Plate Work in general. Dr. Allen will continue the subject of "Contour of the Face." Drs. Rogers, Crane, Hullihen, Brown and Prof. Slack, with many others, have promised to be regular contributors to the Register. We refer to this so that the Profession and the public generally may understand more fully the nature of the work. The time alotted for getting out the first No. was not sufficient to get it up with that taste and in the style we desired, and half the time since the meeting of the Society has been spent some distance from the city, in attendance on an afflicted parent. These circumstances, with the want of experience and the necessary inability of my colleague to afford much assistance at so early a day, will, we hope, be sufficient excuse for any inaccuracy of style or want of proper arrangement that may appear in this.

